# Enteral vitamin A for reducing severity of bronchopulmonary dysplasia in extremely preterm infants: a randomised controlled trial

**DOI:** 10.1186/s12887-017-0958-x

**Published:** 2017-12-16

**Authors:** Abhijeet Rakshasbhuvankar, Sanjay Patole, Karen Simmer, J. Jane Pillow

**Affiliations:** 1 King Edward Memorial Hospital, 374 Bagot Road, Subiaco, WA 6008 Australia; 2 Centre for Neonatal Research and Education, Division of Paediatrics and Child Health (M561), Medical School, University of Western Australia, Crawley, WA 6009 Australia; 3 School of Human Sciences (M309), University of Western Australia, Crawley, WA 6009 Australia

**Keywords:** Bronchopulmonary dysplasia, Chronic lung disease, Vitamin A, Preterm infant, Randomized controlled trial

## Abstract

**Background:**

Intramuscular vitamin A supplementation decreases the risk of bronchopulmonary dysplasia (BPD) in very-low-birth-weight preterm infants without significant adverse effects. However, intramuscular vitamin A supplementation is not widely accepted because of the discomfort and risk of trauma associated with repeated injections. Enteral vitamin A supplementation has not been studied adequately in the clinical trials. Enterally administered water-soluble vitamin A is absorbed better than the fat-soluble form. We hypothesised that enteral administration of a water-soluble vitamin A preparation will decrease severity of BPD compared with a control group receiving placebo.

**Methods:**

We plan a double-blind randomised placebo-controlled trial at a tertiary neonatal-perinatal intensive care unit. Eligibility criteria include infants born at less than 28 weeks’ gestational age and less than 72 h of life. Infants with major congenital gastrointestinal or respiratory tract abnormalities will be excluded. After parental consent, infants will be randomized to receive either enteral water-soluble vitamin A (5000 IU once a day) or placebo. The intervention will be started within 24 h of introduction of feeds and continued until 34 weeks’ post-menstrual age (PMA).

The primary outcome is severity of BPD at 36 weeks’ PMA. Severity of BPD will be assessed objectively from the right-shift of the peripheral oxyhaemoglobin saturation versus partial pressure of inspired oxygen (SpO_2_-PiO_2_) curve. We require 188 infants for 80% power and 5% significance level based on an expected 20% decrease in the right shift of the SpO_2_-PiO_2_ curve in the vitamin A group (primary outcome) compared with control group at 36 weeks’ PMA, and a 20% attrition rate.

Secondary outcomes will be plasma and salivary concentrations of vitamin A on day 28 of the trial (first 30 infants), lung and diaphragm function, clinical outcomes at 36 week’ PMA or before discharge/death, and safety of vitamin A.

**Discussion:**

BPD poses a significant economic burden on the health-care system. If our study shows that enteral supplementation of water-soluble vitamin A is safe and effective for decreasing the severity of BPD, it will provide the opportunity to further evaluate a simple, globally acceptable preventive therapy for BPD.

**Trial registration:**

ANZCTR; ACTRN12616000408482 (30th March 2016).

**Electronic supplementary material:**

The online version of this article (10.1186/s12887-017-0958-x) contains supplementary material, which is available to authorized users.

## Background

Bronchopulmonary dysplasia (BPD) is a major respiratory morbidity associated with premature birth and affects 41% of the infants born before 28 weeks of gestational age [1]. BPD is associated with significant long-term health-consequences, which may persist to school age and adolescence. Infants with BPD are more likely to have chronic cough and asthma like symptoms in school age, abnormal lung function and lung imaging in adolescence, and greater need for hospitalisation and respiratory morbidity as compared with premature infants of similar gestational age without BPD [2–4]. Even more importantly, BPD may adversely influence long-term neurodevelopmental outcomes [5–7]. The current armamentarium for prevention of BPD includes surfactant, caffeine, lung protective ventilation strategies, and targeted oxygen saturation. BPD remains a heavy burden on healthcare resources despite current integrated approaches to therapy for BPD.

Low plasma and tissue concentrations of vitamin A in very low birthweight (VLBW) infants may contribute to the pathophysiology of BPD [8]. Intramuscular (IM) vitamin A supplementation decreases the incidence of BPD in VLBW infants [9, 10]. However, the practice of IM vitamin A supplementation is not widely accepted because of the discomfort and risk of trauma associated with repeated IM injections [11]. In addition, high cost and limited availability of Vitamin A parenteral preparations may further deter physicians from the use of IM vitamin A for prevention of BPD [12]. The intravenous (IV) route of administration is invasive, difficult to maintain for a long term, and associated with increased risk of infection: hence IV administration of Vitamin A is not suitable for prolonged duration of preventive therapy in preterm infants.

Enteral administration of Vitamin A offers a less-invasive route of administration; however, enteral Vitamin A is not well evaluated in the preterm infant. Two randomised controlled trials (RCT) by Wardle et al. and Calisici et al. did not show a significant beneficial effect of enteral vitamin A supplementation for prevention of BPD [13, 14]. The ineffectiveness of enteral vitamin A supplementation for prevention of BPD may be related to decreased bioavailability of enteral vitamin A in the preterm infant. Nevertheless, the RCT by Calisici et al. was only presented as an abstract and hence provides inadequate details about intervention and outcomes. Similarly, the trial by Wardle et al. was limited by inadequate sample size for the primary outcome of BPD, use of postnatal steroids in a large proportion of the study infants, and use of a low vitamin A dose in infants that were at highest risk of BPD [13, 14].

The exact mechanisms involved in the process of absorption of vitamin A through the gut are unclear. Poor absorption of enteral vitamin A in extremely preterm infants may be related to decreased hydrolysis of retinyl esters, decreased availability of bile salts required for formation of micelles, or inadequate availability of carrier proteins required for absorption of vitamin A in enterocytes [15]. Passive diffusion is the predominant mode of absorption at high intraluminal concentrations of vitamin A [16]. In contrast, protein-mediated transport predominates at lower intraluminal concentrations of vitamin A [16]. The small particle size of Vitamin A in the water-soluble form may be advantageous for improved absorbance by diffusion as compared to the larger particle size of the fat-soluble Vitamin A preparations. The water-soluble form of vitamin A is absorbed better by preterm infants compared with the fat-soluble form [17]. The water versus fat-solubility of vitamin A preparations may be critical to interpretation of randomised trials of enteral vitamin A: Wardle et al. and Calicisi et al. did not report the form of vitamin A used in their RCTs [13, 14]. The NeoVitaA trial is using a fat-soluble form of vitamin A ([18] and personal communication). To our knowledge there are no RCTs investigating enteral supplementation of a water-soluble form of vitamin A for prevention of BPD.

Our objective is to undertake a randomised controlled trial to determine if extremely preterm infants (< 28 w gestation) receiving enteral water soluble Vitamin A supplements from commencement of enteral feeds until 34 w PMA compared to a placebo enteral supplement, will have a reduced severity of BPD at 36 w PMA (Additional file 1). We hypothesize that compared to placebo, enteral supplementation with water-soluble vitamin A will decrease the severity of BPD as measured by right shift in the peripheral oxyhaemoglobin saturation versus partial pressure of inspired oxygen (SpO_2_-PiO_2_) curve [19]. We will define a clinically significant reduction in BPD severity as a 20% decrease in the right shift of the SpO_2_-P_i_O_2_ curve: this change approximates the shift required to change from severe BPD to moderate BPD, moderate BPD to mild BPD, or mild BPD to no BPD (unpublished observations, J Pillow).

A sub-study will evaluate the utility of salivary retinol for assessment of Vitamin A status (Vitamin A plasma-saliva correlation sub-study). Use of saliva for the measurement of hormones and vitamins is gaining attention because of its ease of collection, painless nature and low potential for patient harm. Adult salivary and plasma retinol are correlated strongly [20]. A strong correlation of salivary and plasma retinol in very preterm infants will facilitate development of acceptable and non-invasive assessment of vitamin A status in these infants.

## Methods

### Study design and setting

**Fig. 1:**
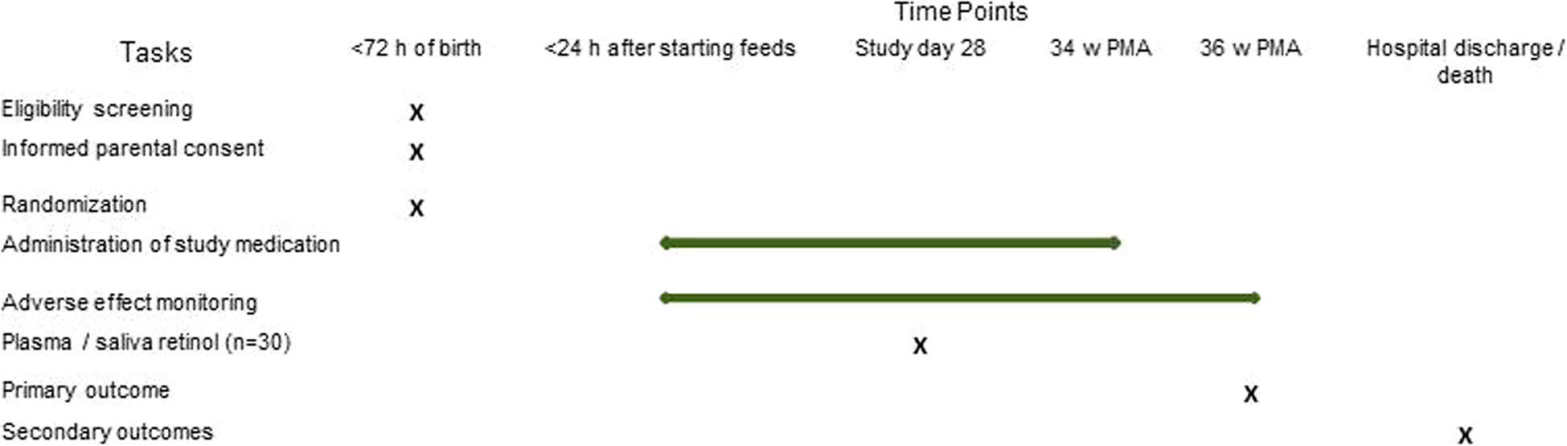
EVARO study schedule. PMA: Post menstrual age

A placebo-controlled double-blind randomised trial (RCT) in a tertiary neonatal intensive care unit. The study schedule is shown in Fig. 1.

Inclusion criteria:

1. Infants born at less than 28 weeks’ gestational age.
2. Less than 72 h after birth.
3. Informed and signed consent from the parents or legal guardian

### Exclusion criteria

Infants with major congenital gastrointestinal or respiratory tracts abnormalities will not be recruited.

Infants admitted in the neonatal intensive care unit will be screened for the eligibility by the chief investigator (AR). AR will approach the parents/legal guardian of the infants and obtain informed consent for the participation in the study.

### Primary outcome

Right shift of SpO_2_-PiO_2_ curve indicates impaired gas exchange and correlates with the severity of BPD using the NICHD classification of BPD severity [19]. The right shift of the SpO_2_-PiO_2_ curve will be assessed at 36 weeks’ (range 35 to 37 week) PMA.

Our decision to use right shift in SpO_2_-PiO_2_ curve as the primary outcome rather than the NICHD categorical classification based on requirement for supplemental oxygen and/or mechanical respiratory support at 36 weeks’ PMA (current clinical BPD severity discriminator) [21] is due to limitations of the NICHD definition: the categorical severity descriptors have limited discriminatory capacity; the SpO_2_ criteria used to prescribe oxygen supplementation vary between clinical units; and the SpO_2_ for any given fractional inspired oxygen (FiO_2_) is affected by altitude of the test site, making it difficult to compare results of two places at different altitude. Reduced alveolar ventilation:perfusion (V_A_/Q) ratio (as assessed using SpO_2_-PiO_2_ curve) is the predominant mechanism of impaired gas exchange in BPD. V_A_/Q ratio can be quantified non-invasively and provides an objective continuous measure of BPD severity, regardless of altitude. Improvement in the V_A_/Q ratio reflects decreased severity of BPD and is detected by decreased right shift of SpO_2_-PiO_2_ curve. Further, the SpO_2_-PiO_2_ curve differentiates oxygen requirement resulting from V_A_/Q mismatch from right-to-left shunt because V_A_/Q mismatch displaces the entire curve to the right while right-to-left shunt lowers the plateau of the curve [19].

#### Measurement of SpO_2_-PiO_2_ curve [22, 23]

BPD severity will be determined at 36 weeks’ PMA or before transfer/discharge if transfer/discharge occurs before 36 weeks’ PMA. The infant’s baseline SpO_2_ will be recorded at the prevailing PiO_2_. PiO_2_ will be incremented or decremented by ~ 2 kPa at 5 min intervals until at least five SpO_2_ measures in the range of 86-97% are recorded (lowest permissible PiO_2_ 14 kPa). Paired measurements of SpO_2_-PiO_2_ are plotted, and the right-shift and V_A_/Q are determined using an algorithm described by Quine et al. [19].

**Secondary outcomes**:

1. Plasma-saliva correlation sub-study (first 30 study infants): Paired saliva and blood samples will be collected to assess correlation between salivary and plasma retinol levels. Saliva (0.25 mL) will be collected using purpose-designed swabs (SalivaBio Infants Swab, Salimetrics™ USA). Paired samples of plasma and saliva will be stored at −80 °C until further analysis. Retinol concentration in the samples will be measured using high performance liquid chromatography with mass spectroscopy [24, 25].

A plasma retinol level > 0.70 μmol·L^−1^ is considered normal while levels of <0.35 μmol·L^−1^ and 0.35 – 0.7 μmol·L^−1^ are considered to be ‘deficient’ and ‘low’ respectively.

2. Relative Dose-Response test (RDR) (30 study infants): During the initial phase of vitamin A deficiency, the plasma retinol concentration remains within normal range at the cost of liver vitamin A stores. The plasma retinol value correlates poorly with liver stores until plasma retinol becomes very low (< 0.35 μmol·dL^−1^). RDR reflects the vitamin A status of an individual better than plasma retinol. The RDR test is based on the principle that apo-retinal binding protein (RBP) accumulates in the liver during vitamin A deficiency. A challenge dose of vitamin A allows retinol to bind to excess hepatic RBP, which is exported into the plasma as the holo-RBP-retinol complex, thereby increasing plasma retinol concentration. The proportional rise in the plasma retinol concentration correlates directly with the severity of liver vitamin A depletion. A RDR value of >20% indicates deficient liver vitamin A stores [26].

The RDR will be measured on day 28 of the trial, 24 h after the previous study dose. Baseline plasma retinol (B_0_) will be estimated from a blood sample (0.5 mL) collected in a lithium heparin tube (BD Microtainer™ Plasma Separator Tube). Capillary, venous or arterial samples are acceptable, as method of collection does not influence plasma retinol values significantly [27]. Whenever possible, the blood sampling will be performed along with the routine blood investigations to avoid additional skin pricks to the infant. The tube will be labelled and wrapped with aluminium foil to protect the sample from light. Plasma obtained by immediate centrifugation at 3000 rpm for 5 min will be stored at −80 °C until further analysis.

After collection of the B_0_ sample, 5000 IU vitamin A (open label) will be administered through a gastric tube to the infant. Five hours after the administration of vitamin A, a second blood (B_5_) sample will be collected and stored using the same technique employed for collection and storage of baseline samples. RDR will be calculated using the formula: Blood RDR = (B_5_ – B_0_) × 100/B_5_

3. Other secondary outcomes measured at discharge or death: Death before discharge; moderate to severe BPD [21]; use of postnatal steroids for BPD; duration of supplemental oxygen; proportion of infants discharged with home oxygen, days of mechanical ventilation; days of positive pressure support (mechanical ventilation + continuous positive airway pressure + humidified high flow); weight gain (gram/day) during the period of study medication supplementation; retinopathy of prematurity requiring treatment in the form of laser ablation or bevacizumab injection [28]; diagnosis of culture positive sepsis (blood or cerebrospinal fluid); diagnosis of suspected sepsis (C-reactive protein >25 mg·L^−1^ and treatment with antibiotics for at least 5 days); grade 3 or 4 intraventricular haemorrhage /periventricular leucomalacia [29]; stage 2a or greater necrotizing enterocolitis [30]; and vitamin A adverse effects.

### Data collection and management

The chief investigator will be responsible for the data collection and management. Deidentified data will be stored in a password protected Research Electronic Data Capture (REDCap) system hosted at King Edward Memorial Hospital [31]. REDCap is a secure, web-based application designed to support data capture for research studies, providing: (1) an intuitive interface for validated data entry; (2) audit trails for tracking data manipulation and export procedures; (3) automated export procedures for seamless data downloads to common statistical packages; and (4) procedures for importing data from external sources [31]. Only authorised persons will have access to the data. The consent forms and associated paperwork available in the hard copy will be stored securely to maintain privacy and confidentiality of research participants. AR, SP, KS and JP will have access to the final data set.

### Sample size

The normal SpO_2_:PiO_2_ curve in adults is shifted to the right of oxygen-haemoglobin dissociation curve by 6 kPa. Preterm infants with moderate to severe BPD have a mean (SD) shift of 16.5 (4.7) kPa [19]. We estimate that up to 20% of the recruited infants may not have SpO_2_-PiO_2_ measurement done at 36 weeks due to death, withdrawal of consent, and transfer of the patient to other hospitals. Allowing for this 20% loss of the cohort before measurement of the primary outcome, the required sample size is 188 (94 in each group) to detect a 20% change in the rightward shift in the treatment group compared with the control (power 80% with two-tailed test, significance 5%). An average of 110 infants less than 28 weeks gestational age are born per year at King Edward Memorial Hospital. All infants admitted in the neonatal intensive care unit will be screened daily to identify eligible infants. We expect to complete the recruitment in less than 3 years.

### Statistical analysis

The data will be analysed statistically based on “intention to treat” using a statistical package (SPSS, version 24.0, IBM Corporation and others, USA). The primary outcome of right shift in the SpO_2_-PiO_2_ curve is a continuous outcome and will be reported as mean ± standard deviation for the intervention and control study groups. The primary outcome will be compared between the two groups using Student’s t test and the result will be reported as “mean difference with 95 % confidence intervals”.

Secondary outcomes will be compared between the two groups using χ^2^ test for categorical data and either the Mann-Whitney U test (not normally distributed) or Student’s t test (normally distributed) for continuous data. The correlation between serum and salivary vitamin A levels, and blood and saliva RDR values will be tested using Pearson r correlation analysis. Bland-Altman analysis will be used to analyse agreement between two assays. A “p” value of <0.05 will be considered statistically significant.

### Randomisation and allocation concealment

The infants will be stratified for randomisation according to the sex and gestational age at birth (23^0^ to 25^6^ and 26 to 27^6^ weeks). Infants will be randomised to treatments by the hospital pharmacist using a computer generated random table (REDCap) in blocks of six within the REDCap randomisation module [31]. The pharmacist will not have any clinical information regarding the recruited infant apart from stratification group and infant sex.

### Blinding

Vitamin A and placebo (normal saline mixed with a safe colouring agent, quinoline yellow) will be dispensed by pharmacy in identical amber coloured containers. It will not be possible to distinguish the medication and the placebo from the appearance, smell and other physical properties. Therefore, medical and nursing staff involved in the care of infant as well as the investigators, will be blinded to the group allocation.

### Intervention

The treatment group will receive oral water-soluble vitamin A (Bio-Logical™ Vitamin A Solution, Biological Therapies, Victoria) containing 5000 IU (0.5 mL) of retinyl palmitate once daily through the gastric tube followed by a feed. In infants receiving continuous milk feeds, the medication will be followed by 0.5 mL normal saline flush before recommencement of the continuous feed. The vitamin A preparation contains pegylated castor oil as an emulsifier to solubilize vitamin A in water [32]. The control group will receive an identical volume (0.5 mL) of placebo solution (normal saline mixed with food colouring agent Quinoline Yellow 2.5 mg/100 mL of normal saline) using the same method described above. Quinoline yellow is a poorly absorbed (< 4%), safe food colouring agent, used in foods, medicines, and cosmetics, and approved for use in Australia, European Union, United States, and Canada [33]. The study medications will be started within 24 h of commencement of enteral feeds and continued until 34 weeks’ PMA.

The dose of vitamin A is same for all the study participants and is not based on weight. This is because smaller infants have lower vitamin A stores and are at higher risk of BPD and hence require larger dosages relative to their body weight. A similar weight independent dose regimen in extremely preterm infants was also used in previous vitamin A supplementation trials [10, 27, 34].

A significant proportion of extremely preterm infants remain vitamin A deficient at 4 weeks of life in spite of Vitamin A supplementation [10, 13]. Vitamin A stores are negatively correlated with gestation. Therefore, lower gestational age infants have increased vitamin A requirements and hence may take longer time to replenish Vitamin A stores. Hence, we planned the duration of supplementation based on the PMA rather than for a fixed number of days.

### Other sources of vitamin a in the study infants

1. Parenteral nutrition: Fat soluble vitamins are added to the lipid emulsion of parenteral nutrition at KEMH. Daily infant intake of vitamin A will be 345 μg·kg^−1^ (966 IU·kg^−1^) whilst on parenteral nutrition with 3 g·kg^−1^·day^−1^ of lipids.
2. Enteral nutrition: Expressed (mother’s) breast milk (EBM) is used preferentially for feeding. If EBM is insufficient to meet the infant’s milk requirements, it is supplemented with pasteurised donor human milk. Milk is fortified once infants are fully enterally fed, to provide 546 μg·kg^−1^·day^−1^ (1820 IU·kg^−1^·day^−1^) of vitamin A when fed at 170 mL·kg^−1^·day^−1^. No additional vitamin A supplement is routinely used at KEMH when infants are receiving fortified human milk.

### Adverse effect monitoring

All infants will be examined by clinical staff daily, and any concerns regarding possible vitamin A adverse effects will be reported immediately to the Chief Investigator. In addition, all the study infants will have weekly physical examination by a neonatologist (AR) for the signs of vitamin A toxicity. Examination findings will be noted in a monitoring chart for the periodic review of the Safety Committee. The examination will include but will not be limited to:

1. Palpation of anterior fontanelle (AF): The AF will be palpated with infant in a quite state and held in the sitting position. The normal AF is flat, flushed with the skin and soft [35]. A tense and bulging AF resulting from benign intracranial hypertension may indicate vitamin A adverse effect. A bulging AF is present in infants with intraventricular haemorrhage and hydrocephalus which are conditions not related to vitamin A toxicity. Hence, the infants with a tense and bulging AF will have cranial ultrasound examination to exclude intraventricular haemorrhage as the cause of bulging fontanelle.
2. Liver size: Liver will be palpated and recorded as distance (cm) below the costal margin in the mid-clavicular line. Liver function tests will be performed if hepatotoxicity is suspected.
3. Skin changes: Skin will be inspected for desquamation, particularly on the palm and sole, and on mouth or lip fissures.

**Reporting of adverse effects:**

1. Safety Committee: The safety committee will comprise a pharmacist, a laboratory scientist and a neonatal consultant. The committee will review adverse effect charts of the study infants periodically after recruitment of 50, 100 and 150 infants. Any serious adverse effects will be reported immediately to the committee.
2. Therapeutic Goods Administration, Australia (TGA) and Ethics Committee: Any serious adverse effect will be reported immediately to TGA and the hospital Human Research Ethics Committee.

### Potential risk

The daily requirement of vitamin A for preterm infants is unknown. Recommended supplementations for the VLBW infants are in the range of 1000 - 1500 IU·kg^−1^d^−1^, regardless of the route of administration [36]. However, higher doses of vitamin A may be warranted in low birth weight infants for prevention of morbidity and mortality [37]. The RCT by Wardle et al. used a daily oral dose of 5000 IU·kg^−1^ in extremely-low-birth-weight infants without significant side effects [13]. Similar doses (5000 IU orally once a day) are used routinely in preterm infants without adverse effects [38]. There was no clinical or biochemical evidence of vitamin A toxicity in the study by Tyson et al., which used 5000 IU vitamin A IM on alternate days for 4 weeks [10]. The Cochrane meta-analysis did not show any significant adverse effects of additional doses of vitamin A to extremely-low-birth-weight infants for prevention of BPD [9]. Thus our proposed enteral dose of 5000 IU per day is safe for extremely preterm infants and is unlikely to be associated with adverse reactions. Vitamin A toxicity is associated with nausea, vomiting, anorexia, pruritus, bulging fontanelles, lack of weight gain, and less commonly pseudotumor cerebri in infants and children. Symptoms of toxicity subside rapidly after withdrawal of the vitamin [39]. In our study, all the study infants will be monitored physically for vitamin A adverse effects.. The study medication will be stopped immediately should Vitamin A toxicity be suspected, and the Safety and the Ethics committee will be informed.

### Dissemination of the trial findings

Results of the trial will be presented in scientific meetings and published in a peer reviewed scientific journal.

## Discussion

The number of preterm births and survival of more premature infants in developed countries is increasing over the last four decades. Nonetheless, BPD remains a major public health problem and contributes to the economic burden of caring for extremely preterm infants. The incremental cost of care for a diagnosis of BPD in preterm infants in 2007 was $31,565 during the initial hospitalisation after birth and $12,472 over the remaining first 2 years of life [40, 41]. An estimated 12,000 infants with BPD are born each year in the United States of America [42]. Thus, the total annual extra expenditure for children <2 years of age with BPD in the United States of America would be $528 million. This amount is probably an underestimate of the overall economic impact of BPD as infants with BPD continue to have greater respiratory symptoms and abnormal lung function through childhood and adolescence. In addition, we expect an increase in the number of preterm infants at risk of developing BPD with improvement in the healthcare facilities in the developing world. Considering the huge economic cost of BPD, more research focusing on prevention of BPD is required. Preventive BPD therapies will be well accepted if they are easy to administer, safe and cost-effective.

IM vitamin A supplementation to VLBW infants decreases risk of BPD. However, the practice is not popular because of the discomfort and risk of trauma associated with the repeated IM injections [11]. If our study shows that enteral supplementation of water-soluble vitamin A is safe, reduces ventilation-perfusion mismatch, and hence severity of BPD as assessed using SpO_2_-PiO_2_ curve, it will pave the way to a multicentre RCT with presence and severity of BPD as a primary outcome.

## Additional file

Additional file 1: SPIRIT (Standard Protocol Items: Recommendations for Interventional Trials). SPIRIT checklist for EVARO study: It gives location of the SPIRIT item description in the manuscript. (DOC 120 kb)

## Abbreviations

AF: Anterior fontanelle
BPD: Bronchopulmonary dysplasia
FiO_2_: Fraction of inhaled oxygen
IM: Intramuscular
PMA: Post-menstrual age
RBP: Retinal binding protein
RCT: Randomised controlled trial
RDR: Relative Dose-Response
SpO_2_: pulse-oximetry oxygen saturation
SpO_2_-PiO_2_: Pulse-oximetry oxygen saturation versus partial pressure of inspired oxygen
V_A_-Q: Ventilation:perfusion
VLBW: Very-low-birth-weight

## References

[1] Gortner L, Misselwitz B, Milligan D, Zeitlin J, Kollee L, Boerch K (2011). Rates of bronchopulmonary dysplasia in very preterm neonates in Europe: results from MOSAIC cohort. Neonatology.

[2] Baraldi E, Filippone M (2007). Chronic lung disease after premature birth. N Engl J Med.

[3] Bhandari A, McGrath-Morrow S (2013). Long-term pulmonary outcomes of patients with bronchopulmonary dysplasia. Sem Perinatol.

[4] Carraro S, Filippone M, Da Dalt L, Ferraro V, Maretti M, Bressan S (2013). Bronchopulmonary dysplasia: the earliest and perhaps the longest lasting obstructive lung disease in humans. Early Hum Dev.

[5] Walsh MC, Morris BH, Wrage LA, Vohr BR, Poole WK, Tyson JE (2005). Extremely low birthweight neonates with protracted ventilation: mortality and 18-month neurodevelopmental outcome. J Pediatr.

[6] Schmidt B, Asztalos EV, Roberts RS, Robertson CMT, Sauve RS, Whitfield MF (2003). Impact of bronchopulmonary dysplasia, brain injury, and severe retinopathy on the outcome of extremely low-birth-weight infants at 18 months. JAMA.

[7] Natarajan G, Pappas A, Shankaran S, Kendrick DE, Das A, Higgins RD (2012). Outcomes of extremely low birth weight infants with bronchopulmonary dysplasia: impact of the physiologic definition. Early Hum Dev.

[8] Shenai JP (1999). Vitamin a supplementation in very low birth weight neonates: rationale and evidence. Pediatrics.

[9] Darlow BA, Graham PJ (2011). Vitamin A supplementation to prevent mortality and short- and long-term morbidity in very low birthweight infants. Cochrane Database Syst Rev.

[10] Tyson JE, Wright LL, Oh W, Kennedy KA, Mele L, Ehrenkranz RA (1999). Vitamin a supplementation for extremely-low-birth-weight infants. N Engl J Med.

[11] Ambalavanan N, Kennedy K, Tyson J, Carlo WA (2004). Survey of vitamin A supplementation for extremely-low-birth-weight infants: is clinical practice consistent with the evidence?. J Pediatr.

[12] Couroucli XI, Placencia JL, Cates LA, Suresh GK (2016). Should we still use vitamin a to prevent bronchopulmonary dysplasia?. J Perinatol.

[13] Wardle SP, Hughes A, Chen S, Shaw NJ (2001). Randomised controlled trial of oral vitamin A supplementation in preterm infants to prevent chronic lung disease. Arch Dis Child Fetal Neonatal Ed.

[14] Calisici E, Yarci E, Degirmencioglu H, Oncel MY, Oguz SS, Uras N (2014). The effects of early oral vitamin A treatment on the prevention of bronchopulmonary dysplasia in low birth weight infants. Arch Dis Child.

[15] Goncalves A, Roi S, Nowicki M, Dhaussy A, Huertas A, Amiot MJ (2015). Fat-soluble vitamin intestinal absorption: absorption sites in the intestine and interactions for absorption. Food Chem.

[16] Reboul E (2013). Absorption of vitamin A and carotenoids by the enterocyte: focus on transport protein. Nutrients.

[17] Morales S, Chung AW, Lewis JM, Messina A, Holt LE (1950). Absorption of fat and vitamin A in premature infants: II effect of particle size on the absorption of these substances. Pediatrics.

[18] Meyer S, Gortner L, NeoVitaA Trial investigators (2014). Early postnatal additional high-dose oral vitamin A supplementation versus placebo for 28 days for preventing bronchopulmonary dysplasia or death in extremely low birth weight infants. Neonatology.

[19] Quine D, Wong CM, Boyle EM, Jones JG, Stenon BJ (2006). Non-invasive measurement of reduced ventilation:perfusion ratio and shunt in infants with bronchopulmonary dysplsia: a physiological definition of the disease. Arch Dis Child Fetal Neonatal Ed.

[20] Saral Y, Coskun BK, Ozturk P, Karatas F, Ayar A (2005). Assessment of salivary and serum antioxidant vitamins and lipid peroxidation in patients with recurrent aphthus ulceration. Tohoku J Exp Med.

[21] Ehrenkranz RA, Walsh MC, Vohr BR, Jobe AH, Wright LL, Fanaroff AA (2005). Validation of the National Institute of health consensus definition of bronchopulmonary dysplasia. Pediatrics.

[22] Svedenkrans J, Wood AJT, Pillow JJ (2015). Predictors of right shift and ventilation/perfusion in very preterm infants. J Paed Child Health.

[23] Svedenkrans J. Consequences of preterm birth on lung function, physical activity and exercise capacity. https://openarchive.ki.se/xmlui/bitstream/handle/10616/45569/Thesis_Jenny_Svedenkrans.pdf?sequence=1. Accessed 27 July 2017.

[24] Su Q, Rowley KG, O’Dea K (1999). Stability of individual carotenoids, retinol and tocopherols in human plasma during exposure to light and after extraction. J Chromatogr B Biomed Sci Appl.

[25] Craft NE, Brown ED, Smith JC (1988). Effects of storage and handling conditions on concentrations of individual carotenoids, retinol, and tocopherol in plasma. Clin Chem.

[26] Tanumihardjo SA. Biomarkers of vitamin A status: what do they mean? In: World Health Organization. Report: Priorities in the assessment of vitamin A and iron status in populations, Panama City, Panama, 15–17 September 2010. Geneva: World Health Organization; 2012.

[27] Kennedy KA, Stoll BJ, Ehrenkranz RA, Oh W, Wright LL, Stevenson DK (1997). Vitamin A to prevent bronchopulmonary dysplasia in very-low-birth-weight infants: has the dose been too low?. Early Hum Dev.

[28] An International Committee for the classification of Retinopathy of prematurity (2005). The international classification of retinopathy of prematurity revisited. Arch Ophthalmol.

[29] Volpe JJ (1977). Neonatal intracranial haemorrhage. Pathophysiology, neuropathology, and clinical features. Clin Perinatol.

[30] Bell MJ (1978). Neonatal necrotizing enterocolitis. N Engl J Med.

[31] Harris PA, Taylor R, Thielke R, Payne J, Gonzalez N, Conde JG (2009). Research electronic data capture (REDCap) – a metadata-driven methodology and workflow process for providing translational research informatics support. J Biomed Inform.

[32] Vitamin A Solution http://www.biologicaltherapies.com/node/391 Accessed on 28 July 2016.

[33] European Food Safety Authority (2009). Scientific opinion on the re-evaluation of Quinoline yellow (E104) as a food additive. EFSA J.

[34] Ambalavanan N, Wu TJ, Tyson JE, Kennedy KA, Roane C, Carlo WA (2003). A comparison of three vitamin A dosing regimens in extremely-low-birth-weight infants. J Pediatr.

[35] Johnson L, Cochran WD, Cloherty JP, Eichenwald EC, Hansen AR, Stark AR (2012). Assessment of the newborn history and physical examination of the newborn. Manual of neonatal care.

[36] Bolisetty S, Osborn D, Sinn J, Lui K, the Australasian Neonatal Parenteral Nutrition Consensus Group (2014). Standardised neonatal parenteral nutrition formulations – an Australasian group consensus 2012. BMC Pediatr.

[37] Koletzko B, Goulet O, Hunt J, Krohn K, Shamir R, for the Parenteral Nutrition Guidelines Working Group (2005). Guidelines on paediatric parenteral nutrition of the European society of paediatric gastroenterology, hepatology and nutrition (ESPGHAN) and the European society for clinical nutrition and metabolism (ESPEN), supported by the European society of paediatric research (ESPR). J Ped Gastroenterology Nutrition.

[38] Landman J, Sive A, Heese HDV, Elst CVD, Sacks R (1992). Comparison of enteral and intramuscular vitamin A supplementation in preterm infants. Early Hum Dev.

[39] Bendich A, Langseth L (1989). Safety of vitamin a. Am J Clin Nutr.

[40] Null DM, D’Souza AO, O’Day KB, Happe LE (2008). The economic burden of bronchopulmonary dysplasia during the first two years of life. Arch Dis Child.

[41] Johnson TJ, Patel AL, Jegier B, Engstrom JL, Meier P (2013). The cost of morbidities in very low birth weight infants. J Pediatr.

[42] American Lung Association Lung Disease Data. 2008. https://commed.vcu.edu/Chronic_Disease/Lung/LDD08.PDF. Accessed 15 Sept 2016.

